# Mesothelioma mortality in Europe: impact of asbestos consumption and simian virus 40

**DOI:** 10.1186/1750-1172-1-44

**Published:** 2006-11-07

**Authors:** Katharina Leithner, Andreas Leithner, Heimo Clar, Andreas Weinhaeusel, Roman Radl, Peter Krippl, Peter Rehak, Reinhard Windhager, Oskar A Haas, Horst Olschewski

**Affiliations:** 1 Department of Pulmonology, University Clinic of Internal Medicine, Medical University Graz, Graz, Austria; 2 Department of Orthopedic Surgery, Medical University Graz, Graz, Austria; 3 Molecular Diagnostics, ARCS Seibersdorf, Seibersdorf, Austria; 4 Department of Oncology, University Clinic of Internal Medicine, Medical University Graz, Graz, Austria; 5 Division of Biomedical Engineering and Computing, Department of Surgery, Medical University Graz, Graz, Austria; 6 Children's Cancer Research Institute (CCRI), St. Anna Children's Hospital, Vienna, Austria

## Abstract

**Background:**

It is well established that asbestos is the most important cause of mesothelioma. The role of simian virus 40 (SV40) in mesothelioma development, on the other hand, remains controversial. This potential human oncogene has been introduced into various populations through contaminated polio vaccines. The aim of this study was to investigate whether the possible presence of SV40 in various European countries, as indicated either by molecular genetic evidence or previous exposure to SV40-contaminated vaccines, had any effect on pleural cancer rates in the respective countries.

**Methods:**

We conducted a Medline search that covered the period from January 1969 to August 2005 for reports on the detection of SV40 DNA in human tissue samples. In addition, we collected all available information about the types of polio vaccines that had been used in these European countries and their SV40 contamination status.

**Results:**

Our ecological analysis confirms that pleural cancer mortality in males, but not in females, correlates with the extent of asbestos exposure 25 – 30 years earlier. In contrast, neither the presence of SV40 DNA in tumor samples nor a previous vaccination exposure had any detectable influence on the cancer mortality rate in neither in males (asbestos-corrected rates) nor in females.

**Conclusion:**

Using the currently existing data on SV40 prevalence, no association between SV40 prevalence and asbestos-corrected male pleural cancer can be demonstrated.

## Background

Asbestos is a potent carcinogen and the most important single cause of mesothelioma, a mostly fatal cancer of the pleura [1-3]. Only about 20% of mesothelioma cases occur in non-exposed individuals [4,5]. Previous studies have shown that mesothelioma mortality rates correlate with past asbestos consumption rates (defined by production minus export plus import) in industrialized countries [1,6]. During the past 40 to 50 years, asbestos consumption varied considerably in European countries, with low *per capita *use in less industrialized countries, such as Bulgaria, and vast use in ship building and other insulating industries,*e.g. *in the U.K. Since the time lag between asbestos exposure and tumor development is 30 to 45 years in most cases, the recent increase in mesothelioma incidence should therefore reflect the intensified use of asbestos during this particular period [3,7-9]. Indeed, the production of asbestos peaked worldwide in the late 1970s and early 1980s [10]. Likewise, the mesothelioma incidence is expected to reach maximum levels between 2010 and 2020 in industrialized countries [6]. About half of the cases will occur in construction and shipbuilding workers, as in these professions asbestos exposure was particularly common [6]. Incidence is much lower in women, as they were generally, not involved in asbestos-related activities [11].

The fact that traces of simian virus 40 (SV40) were repeatedly demonstrated in a significant proportion of mesothelioma samples led to the notion that this virus may act as either a co-carcinogen or tumor promoter [12-14]. SV40 was introduced unintentionally into millions of people *via *contaminated poliomyelitis virus vaccines between 1955 and 1963. However, some vaccines produced later may have not been entirely SV40-free, as evidenced by the fact that SV40 DNA has been recently detected in archival polio vaccines produced in 1966 and 1969 by a major Eastern European manufacturer [15]. SV40 was present in both the attenuated (oral) polio vaccine (OPV) and the inactivated polio vaccine (IPV), since formaldehyde treatment, which was used to inactivate the poliomyelitis virus, failed to inactivate SV40 [12].

SV40 DNA was subsequently detected in human brain and bone tumors as well as lymphoma samples (reviewed in [16]). Intriguingly, SV40 causes the same tumor types in hamsters [4]. The most important step in the process of carcinogenesis is the inactivation of tumor suppressor p53 and members of the retinoblastoma family of proteins through the SV40 large T antigen [17]. In addition, other tumor suppressor genes become methylated and are shut down. These gene modifications have not only been observed *in vitro*, but also in an analogous fashion in SV40-positive lymphoma samples [18].

However, it is important to note that SV40 DNA was not detected in several studies of tumor samples from particular populations, whereas it was readily detectable in appropriate control samples from the USA [19,20]. In particular, SV40 DNA was not detected in mesothelioma, brain tumor and bone tumor samples from Austria, Finland and Turkey, countries which apparently had never used contaminated polio vaccines [19-24]. These findings were therefore taken as evidence that the population-specific and linked geographic differences were genuine and that they reflected the heterogeneous use of SV40-contaminated polio vaccines in the respective countries [4]. However, definitive epidemiological proof for the presence or absence of an association between (past) SV40 exposure and cancer is lacking so far, mostly because the infected cohorts can no longer be identified unambiguously [4].

The presence of virus-specific antibodies in serum is a well-established biomarker of viral infection. Antibodies to SV40 have been measured in humans by plaque inhibition neutralization assays and enzyme immunoassays. However, the studies have been mostly negative or detected only low levels of SV40 serum antibodies [25-28]. Cross-reactivity between SV40 and the BK polyomavirus has been proposed as an explanation for the detection of low levels of SV40-reactive antibodies in human serum samples and may complicate the interpretation of positive assay results [27]. In a recent population-based case-control study published by Engels *et al. *[26] using competitive assays to analyze SV40-specific reactivity, it has been shown that an estimate of 1% to 1.6% individuals from the U.S. born before 1963 had SV40 specific antibodies, whereas those born later showed no SV40 specific reactivity. The authors stated that these results point to the possibility that exposure to SV40 could have led to antibody responses that declined over the decades, probably due to lack of virus replication, and that SV40 seems not to be a common cause of infection in humans [26].

However, the past exposure of humans to SV40 is undoubted. We therefore set out to investigate whether mesothelioma (pleural cancer) incidence and mortality rates might be higher in those European countries (including the former Communist countries), in which at least some circumstantial evidence indicates that a population-wide exposure had taken place. For this purpose, we first investigated to what extent pleural cancer mortality rates correlate with those of past asbestos use. We then compared country-specific asbestos consumption and mesothelioma rates with the appropriate likelihood of SV40 contamination. To ascertain whether the respective SV40 exposure was frequent, low or absent in specific countries, we used data from publications that dealt with the analysis of SV40 DNA in human tissue samples. In addition, we also collected all the available information about which type of poliomyelitis virus vaccines had been used in these countries and whether they were SV40-contaminated or not.

## Methods

### Study design

We conducted a Medline search for the period from January 1969 to August 2005 with use of the following terms and Boolean operators: (("SV40" OR "simian virus 40") AND ("tumor" OR "tumour" OR "cancer")) or (("SV40" OR "simian virus 40") AND (" [country]" OR " [country language]")) or (("SV40" OR "simian virus 40") AND ("Europe*")). All abstracts were checked for valid results on SV40 DNA in human tissue samples or for information about past poliomyelitis virus vaccination programs in European countries. The bibliography of each paper was then further screened by two researchers for additional relevant studies.

### Inclusion and exclusion criteria for molecular genetic studies

Articles were independently examined in detail by two investigators (A.L. and K.L.). All studies on SV40 in human tissue samples (mostly tumors) or body fluids derived from a European country were considered for analysis. Eligibility criteria: Only original articles on SV40 detection were included. The origin of the samples had to be clearly stated. As an inclusion criterion, SV40 nucleic acid hybridization or polymerase chain reaction (PCR) methods had to be performed for SV40 detection. Studies on cell lines were not included. One study was excluded because the SV40-positive patient was the newborn child of foreign guest workers [29]. In one study from Berlin, the origin of the patients was not clear (Western or Eastern Germany), the study was therefore excluded [30]. One study with data on SV40 DNA in sewage from two European cities was excluded from the analysis [31]. Although SV40 may be shed in stool [32] the implications of these data are unclear since comparable studies are missing. The relevant data from of the eligible studies were abstracted by one researcher and rechecked for accuracy by another. In particular, we extracted the following information: origin of the tissue specimen, tumor or non-tumor tissue, type of tumor, number of SV40-positive samples, number tested, and detection method.

### Inclusion and exclusion criteria for reports on SV40 in vaccines

All reports retrievable from the Medline search or from the bibliography of each paper containing data on SV40 contamination of vaccines used in a specific European country were eligible for analysis.

### Mesothelioma mortality data

In the absence of a specific International Classification of Diseases [ICD] code for mesothelioma until the introduction of a specific code for mesothelioma (C45) in the 10^th ^revision of the ICD in 1992 [33], pleural cancer (ICD 163) death rates were used for the analysis. Mortality of pleural cancer is mainly attributed to mesothelioma [34] and corresponds reasonably with mesothelioma incidence, since most patients diagnosed with mesothelioma will die within one year [3]. For each country, age-standardized (world standard population) pleural cancer mortality rates were extracted from the World Health Organization (WHO) database [35]. Mortality data from 1985 to 1989 were used because of the unification or division of some European countries after the end of the Cold War (about 1989), in order to allow correlation with former country-wide asbestos consumption data. To allow comparability, the WHO database [35] was the only source of pleural cancer mortality/incidence data used. Cancer incidence rates are known to have been underestimated until 1992 in Belgium (DeVuyst, Hopital Erasme, Brussels, personal communication and [36]), therefore, in the case of Belgium, pleural cancer mortality from 1995 to 1999 was retrieved from the WHO mortality database [33] and was compared to asbestos consumption in 1970 in Belgium-Luxembourg. Since data from Turkey were not retrievable from the WHO database [35], Turkish mesothelioma incidence in 1996, as reported by Menintas *et al. *[37], was correlated with asbestos consumption in 1970.

### Asbestos consumption data

Asbestos consumption data were provided by the U.S. Geological Survey, Reston, VA [10]. For some countries, the information about either asbestos or pleural cancer mortality was unfortunately incomplete: these data were therefore excluded from further analysis.

### Statistical analysis

For statistical analyses, the NCSS 2001 (NCSS Statistical Systems, Kaysville, UT, USA) software package was used. *P *< 0.05 was considered to be significant. The Spearman's correlation coefficient was calculated for the following variables: male/female pleural cancer mortality and asbestos consumption. ANCOVA was used for group comparisons with past asbestos consumption as a covariate, and male/female pleural cancer mortality as a dependent variable. No adjustment for multiple testing was done.

## Results

### Mesothelioma and asbestos

**Figure 1:**
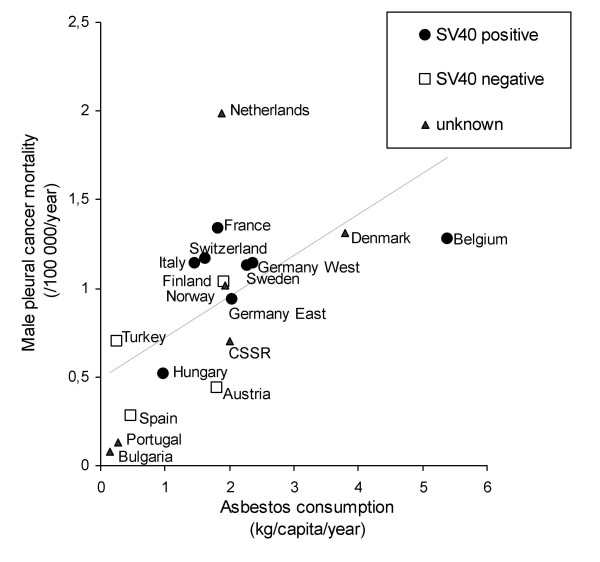
Correlation between *per capita *asbestos consumption and male pleural cancer mortality rates (R = 0.603, *P *= 0.008). In the legend SV40 detection refers to SV40 nucleic acid detection at a cut-off level of 10%. *Per capita *asbestos consumption in a state was calculated as the production plus imports minus exports of all types of asbestos (Data from U.S. Geological Survey).

**Figure 2:**
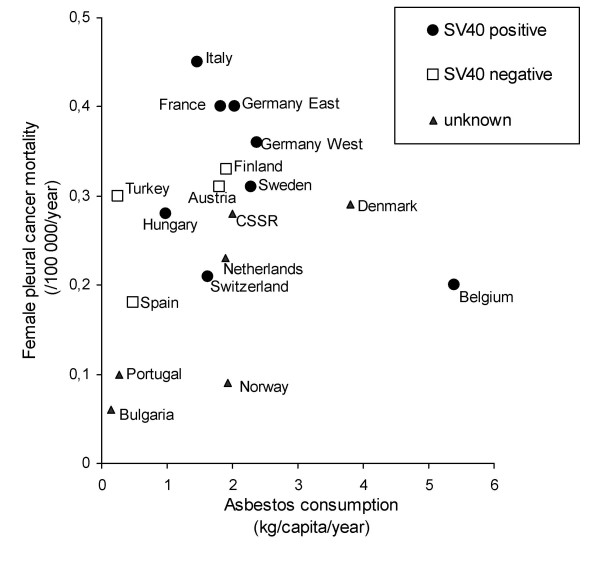
*Per capita *asbestos consumption and female pleural cancer mortality rates in European countries. In the legend SV40 detection refers to SV40 nucleic acid detection at a cut-off level of 10%.*Per capita *asbestos consumption in a state was calculated as the production plus imports minus exports of all types of asbestos (Data from U.S. Geological Survey).

When we correlated male pleural cancer death rates in 18 European countries with *per capita *asbestos consumption 25 to 30 years earlier, we found a linear relationship (Figure 1). The Spearman's correlation coefficient of the two variables is R = 0.603 (*P *= 0.008). Notably, all European countries with available information on asbestos consumption and pleural cancer mortality were included in the analysis (the sources are indicated in the methods section). For females, no linear relationship could be demonstrated (Figure 2). The Spearman's correlation coefficient of the two variables is R = 0.293 (*P *= 0.239).

### SV40 data

**Figure 3:**
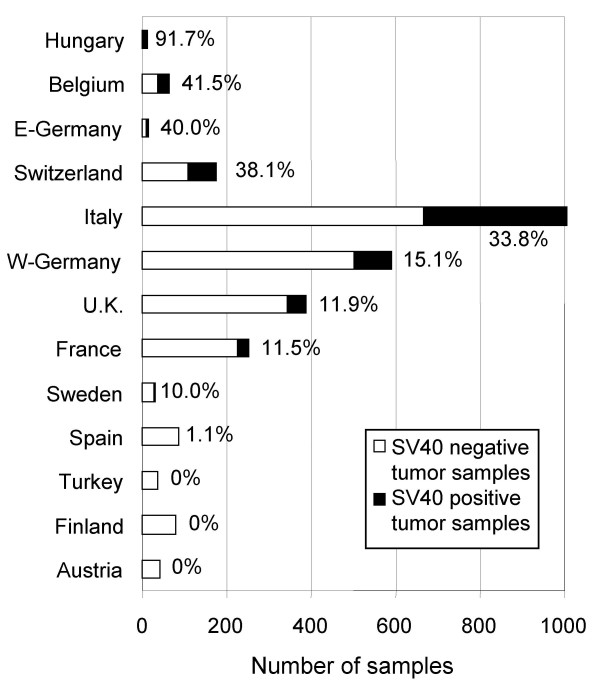
Frequencies of SV40 nucleic acid detection in European human tissue samples. Only reports using polymerase chain reaction or hybridization techniques were included. The original reports are cited in Table 1. Pleural cancer mortality data were not available for the entire United Kingdom, which was therefore excluded from statistical analysis.

**Figure 4:**
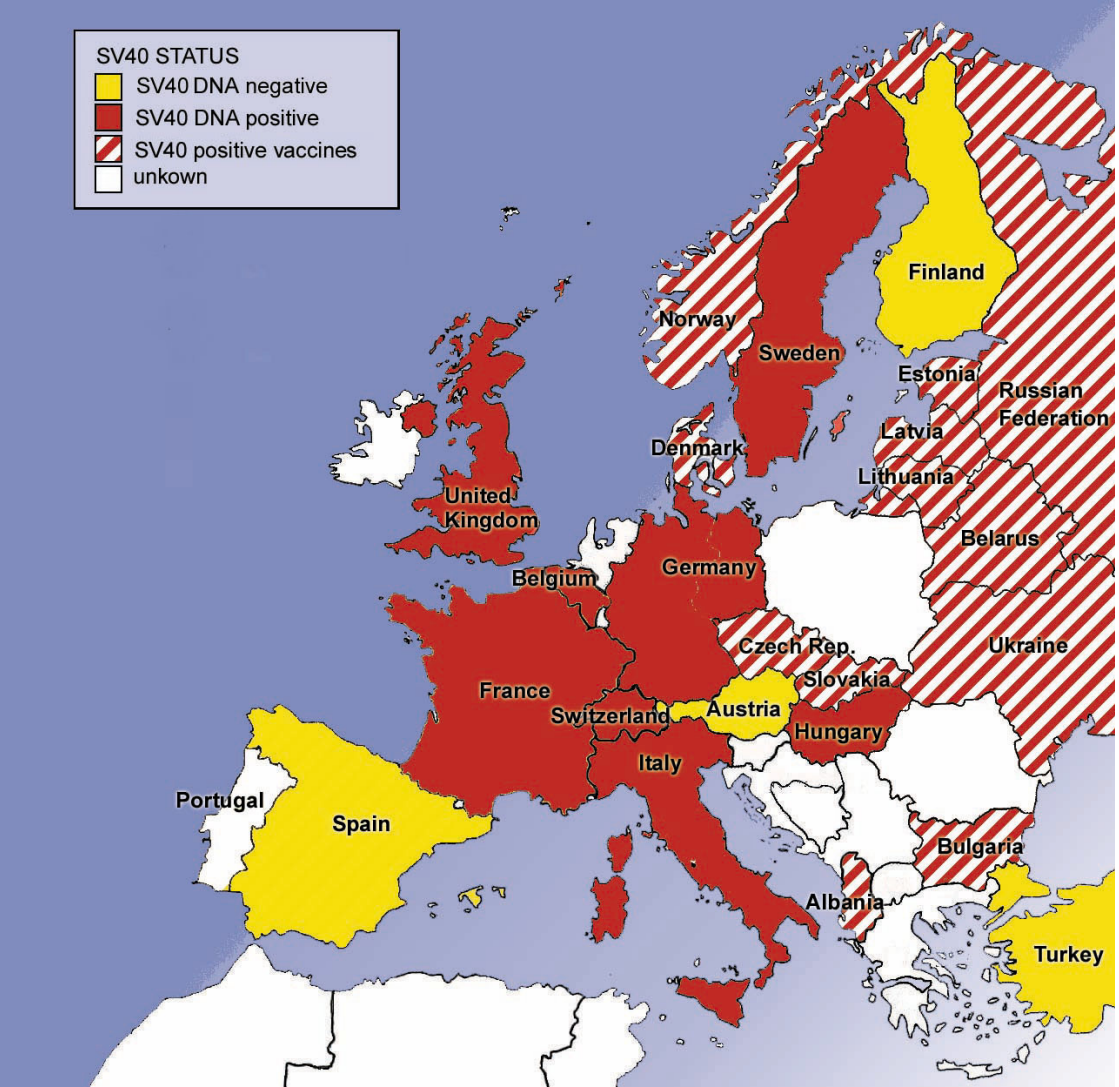
Map of SV40 nucleic acid detection and historical vaccine contamination with SV40 in European countries. For nucleic acid detection a cut-off level of 10% (SV40 positive samples from a country of total examined samples) was chosen.

According to our criteria, 55 original articles on SV40 in human tissue samples or body fluids derived from 13 European countries contained information relevant to our analysis (Figures 3 and 4, Table 1). For all countries except the U.K., data for pleural cancer mortality and past asbestos use were available. Thus, 12 countries were included in the statistical analysis. The cut off level for SV40 detection was set at 0% or 10% positive tumor samples, respectively (Figure 3). Before analyzing the effect of SV40 prevalence, we corrected male pleural cancer rates for asbestos consumption because these two variables are highly correlated. However, whether SV40 DNA had been detected in tumor samples from a particular country or not, had no effect on pleural cancer mortality rates at either cut-off level, neither in males (asbestos-corrected rates) nor in females (Table 2).

**Table 1:** SV40 nucleic acid detection in human samples

Country	*Positive *reports on SV40 nucleic acids	*Negative *reports on SV40 nucleic acids	SV40 detection at 0% cut-off level	SV40 detection at 10% cut-off level
Austria		[21,22]	-	-
Belgium	[38,39]	[40,41]	+	+
Finland		[19,23]	-	-
France	[42]	[41,43]	+	+
Germany West	[44-51]	[52-54]	+	+
Germany East	[55]		+	+
Hungary	[51]		+	+
Italy	[14,17,47,56-70]^a^	[71-73]	+	+
Spain	[74] low positive	[71,75]^b^	+	-
Sweden	[76]		+	+
Switzerland	[77] data also presented in [23]		+	+
Turkey		[20,24]	-	-
United Kingdom^c^	[78-81]	[82-85]	+	+

Total number of studies	37	18		

Percent cut-off level refers to percentage of SV40-positive human tissue samples in all samples tested in a country.

^a^Healthy subjects were tested in [70].

^b^One study [75] contained only uterine cervix carcinoma samples.

^c^Pleural cancer mortality data were available only for England and Scotland, but not for the entire U.K., therefore the U.K. was excluded from further statistical analysis.

**Table 2:** Comparison of pleural cancer mortality rates in countries with or without molecular genetic evidence of SV40

Category	Cut-off level	Mean pleural cancer mortality in SV40-nucleic acid *positive *countries (/100 000/yr)	Mean pleural cancer mortality in SV40-nucleic acid *negative *countries (/100 000/yr)	*P*
Males*	0%	0.97 (n = 9)	0.81 (n = 3)	0.465
Females	0%	0.31 (n = 9)	0.31 (n = 3)	0.956
Males*	10%	1.04 (n = 8)	0.69 (n = 4)	0.082
Females	10%	0.33 (n = 8)	0.28 (n = 4)	0.397

Cut-off level refers to the percentage of SV40-positive human tissue samples of all samples tested in a country. *Asbestos-corrected rates.

### Poliomyelitis virus vaccines

Information regarding the type of poliomyelitis virus vaccine (and whether it had been SV40-contaminated or not) was eligible from 15 countries (Table 3). In ten countries, the usage of SV40-contaminated polio vaccines is unambiguously documented, while in three other countries SV40-contaminated vaccines had apparently not been used (Table 3, Figure 4). For Spain and Poland, contradictory reports exist (Table 3). For ten countries with either positive or negative SV40 contamination of vaccines, data on asbestos consumption and pleural cancer were available (see Figure 1, sources are indicated in the methods section). Our statistical analyses revealed that whether the polio vaccine was contaminated or not, had no impact whatsoever on male asbestos consumption-corrected or female pleural cancer rates. In males, the mean asbestos-corrected mortality rate was 0.77/100 000 (n = 7) in countries with SV40-contaminated vaccines and 0.83/100 000 (n = 3) in countries without SV40-contaminated vaccines (*P *= 0.700). In females, the mean mortality rate was 0.24/100 000 (n = 7) in countries with SV40-contaminated vaccines and 0.31/100 000 (n = 3) in countries without SV40-contaminated vaccines (*P *= 0.377).

**Table 3:** SV40 in poliomyelitis virus vaccines in European countries

Country	Rating of contamination of polio vaccines with SV40	Vaccines, vaccination programs and origin of vaccines
Albania	Positive	Contaminated Russian vaccine (OPV) used since 1960 [86-90].
Austria	Negative	Mass vaccinations with SV40-free British vaccine (OPV) since winter 1961/62 [91-92].
Bulgaria	Positive	Contaminated Russian vaccine (OPV) used since 1960 [86-90].
CSSR	Positive	Since 1960: limited use of IPV, mass vaccinations with OPV, partly with contaminated Russian vaccine [86-90,93-94].
Denmark	Positive	Vaccinations from 1955 with widely contaminated Danish vaccine (IPV), SV40-free from 1963 [95]. A combined schedule was introduced in 1968 [96].
Finland	Negative	Mass vaccinations since 1957 with SV40-free Belgium vaccine (IPV) [19]. Finland has never used OPV on a routine basis [96].
Germany East	Positive	Contaminated Russian vaccine (OPV) used since 1960 [55, 87-90, 97].
Hungary	Positive	Since 1957: limited use of IPV, mass vaccinations with vaccines from the US, Canada, Hungary and Russia (also OPV) [87-90].
Norway	Positive	Vaccinations started 1956 with Danish vaccine (IPV); since 1957 potentially contaminated U.S. vaccine (IPV) [98], change to OPV from 1967 to 1979, then back to IPV from 1979 onwards [96].
Poland	Unclear	Mass vaccinations (OPV) since 1958 with Koprowski strain live vaccine [94]; vaccine was claimed to be Russian made [99], but Russian vaccines were derived from Sabin's strain [86].
Russia (USSR)	Positive	Mass vaccinations since 1959 with contaminated Russian vaccine (OPV). A small proportion of persons were vaccinated with IPV at the beginning of the mass vaccinations. [86-90, 100].
Spain	Unclear	Mass vaccinations since 1963 with British vaccine (OPV) [101]; British vaccines were SV40-free since 1962 [102]; in contrast some vaccines were later claimed to have been contaminated [103].
Sweden	Positive	In 1957 potentially contaminated U.S. vaccine (IPV), from 1958 SV40-free Swedish vaccine (IPV). Sweden has never used OPV [96].
Turkey	Negative	Vaccination was not started before 1970, at a time where polio vaccines were required to be SV40-free [20, 24]. The type of the vaccine is unclear. In a global poliomyelitis eradication initiative starting in 1989, OPV was used.
United Kingdom	Positive	Vaccination started in 1956 with OPV [104-105]. SV40-free since 1962 [102].

CSSR: Czechoslovak Socialist Republic; OPV: oral polio vaccine; IPV: inactivated polio vaccine.

In addition, we analyzed the impact of the type of vaccine (IPV or OPV) used between 1957 and 1963 on pleural cancer rates in Europe. In two of the ten countries (Sweden and Finland), IPV was the only vaccine used at least until 1996, in two other countries (Denmark and Norway) OPV was used as well as IPV, but not before 1967, when Western European vaccines were SV40 free. In four countries, OPV was used between 1957 and 1963, together with variable exposure to IPV. In one country (Hungary) the predominant type of vaccine used between 1957 and 1963 is unclear, and in one country (Turkey) apparently no vaccine has been used between 1957 and 1963. In countries with past use of contaminated IPV, the mean male asbestos-corrected pleural cancer rate was 0.95/100 000 (n = 3), and was 0.77/100 000 (n = 5) in all other countries (*P *= 0.381). In countries with past use of contaminated OPV, the mean male asbestos-corrected pleural cancer rate was 0.77/100 000 (n = 3) and was 0.87/100 000 (n = 5) in all other countries (*P *= 0.619). In countries with past use of contaminated IPV, mean female pleural cancer rate was 0.23/100 000 (n = 3) and was 0.28/100 000 (n = 5) in all other countries (*P *= 0.636). In countries with past use of contaminated OPV, mean female pleural cancer rate was 0.25/100 000 (n = 3) and was 0.27/100 000 (n = 5) in all other countries (*P *= 0.844). Therefore, we did not find any significant differences in pleural cancer rates from countries with past use of SV40-contaminated IPV or OPV compared to the other European countries.

## Discussion

The main purpose of this ecological analysis was to explore the potential effect of a country-wide presence of SV40 on the respective pleural cancer mortality rate, presuming that SV40 has the long adjudicated tumor-inducing or -promoting role in humans. A country-wide presence of SV40 was inferred from previous vaccination programs with contaminated vaccines or the detection of virus DNA in tumor samples. Although the results of our analyses confirmed the previous well-established association between the male pleural cancer mortality rate and the extent of the asbestos consumption 25 to 30 years earlier, we failed to detect any discernible effect on the respective male or female pleural cancer mortality rates that could have been allocated to SV40.

The oncogenic potential of SV40 and the mode of its introduction into the human population *via *contaminated vaccines are well documented (for review see [12]). However, whether viral infection indeed also represents a risk factor for tumor development in humans is still a rather controversial issue, which at present is primarily supported by spurious molecular genetic studies, but not by firm epidemiological evidence [106]. However, even the question of whether SV40 is actually present in human tumors and if so, what role it might play in tumor development, is currently unsolved and a matter of ongoing debate, especially since two multi-center studies reached different conclusions [107,108]. Epidemiological studies about tumors developing in recipients of SV40-contaminated vaccines are mainly hampered by the fact that the infected cohorts are difficult to identify, because SV40 has been found in tumors of patients that were too young to have been exposed to contaminated vaccines [4].

To differentiate between SV40-contaminated and SV40-free European countries, we collected all eligible studies dealing with this topic and extracted the relevant information. However, we excluded all studies which had merely used immunological and serological detection procedures, because SV40 and the human BK and JC polyomaviruses are known to cross-react to a high degree [109-113]. For the same reason, we also excluded reports that dealt with serum antibodies against SV40. We were nevertheless able to identify three countries (Austria, Finland and Turkey), in which exclusively SV40-free polio vaccines had been used. Incidentally, these were the same countries in which molecular genetic studies failed to detect SV40 DNA in a representative large number of tumor samples [19-24].

Although we were unable to find any epidemiological evidence for a potential association between SV40 and pleural cancer, the following limitations of our analysis have to be taken into consideration. First of all, we were unable to account for different types and particular usage of asbestos. Instead, we used the asbestos data from a single source, the U.S. Geological Survey [10]. Naturally occurring asbestos is found in soils or rocks in certain villages in southeast Turkey, where it causes endemic occurrence of mesothelioma [114], and also in other parts of the world, *e.g. *in California [115]. However, it was not possible to correct for the impact of natural asbestos deposits in the cohorts used in this study. Even if Turkey is excluded from the analysis, no significant association between SV40 and pleural cancer is found, neither in males (asbestos-corrected rates), nor in females (data not shown).

Second, differences in cancer registration exist and increasing diagnostic awareness in the past 20 years may have affected the reported mortality rates. Miscoding might have occurred in countries where only death certificates were used for mesothelioma registration [34]. To optimize comparability, we therefore used the WHO database as our only source of information. In the majority of cases, we used mortality data from 1985 to 1989. More recent mortality rates from the WHO mortality database would not allow comparability of mortality rates with former country-wide asbestos consumption data because of the unification or division of certain European countries after the end of the Cold War (about 1989). Data from the International Agency for Research on Cancer (IARC) database [116] were not suitable, because for many countries the incidence rates are indicated on a district-basis only, and not in a country-based fashion. However, the important limitation of possibly incorrect pleural cancer rates could not be overcome in the current study. Therefore, improvements in cancer registration are important for future studies addressing these questions.

Third, methodological differences in SV40 screening procedures (*e.g. *different PCR primers, frozen versus paraffin embedded tissues) lead to unequal sensitivities [117]. An unknown proportion of positive SV40 DNA results may have been related to contamination or to other problems with the PCR methods [118]. Optimized laboratory procedures for SV40 detection have been only recently defined [119]. However, as is suggested, mostly by the laboratory investigators themselves, there might be a relationship between country-specific PCR results and geographic variations in SV40 occurrence [4] and hence mesothelioma incidence. Therefore, to study geographic variations, we chose to assume the accuracy of the prior positive PCR results. The different sample sizes of the SV40 detection studies were taken into account by assessing the number of samples with positive SV40 nucleic acid detection versus the whole number analyzed samples for each eligible study. Further issues that also have to be considered in such analyses are, of course, that potential but yet undefined specific genetic factors might influence the likelihood of mesothelioma development in different populations [120].

In the United States, a potentially contaminated poliomyelitis virus vaccine was used between 1955 and 1963 nationwide on about 90% of children and 60% of adults [16,121]. In Europe, approximately 60% of the population received a potentially contaminated vaccine [121]. However, the SV40 prevalence in many European countries is more mosaic-like, because it still mirrors the pattern of when and with which vaccine mass vaccination programs were initiated by local governments. At the beginning of our investigation, we were confident that we could obtain the necessary SV40 data from the vaccine producing companies, which would have been the most efficient and accurate way of determining the pattern of use of contaminated vaccines. However, many of our requests remained unanswered and in some instances the relevant information had apparently already been destroyed, unclear statements were frequent. Therefore, national health bulletins (like those issued in Austria and Spain) became a particularly important resource [91,92,101]. Reports on historical SV40 contamination of vaccines may be of varying accuracy, depending on the detection methods used and on the number and source of samples (batches) tested. However, all data were assumed to be of equal weight because weighting of such heterogenous data was not possible. Moreover, other routes of transmission may exist. Epidemiological evidence suggests that SV40 may be contagiously transmitted in humans by horizontal infection, independent of the earlier administration of SV40-contaminated polio vaccines [113]. Recently, even infection through laboratory strains of SV40 has been proposed [122].

Although little is known about the severity of SV40 infection in humans (systemic or non-systemic), some data indicate that there might be biological differences depending on the type of exposure (IPV *vs. *OPV). After oral administration of SV40 in humans, there was no antibody response, indicating that it most likely did not result in systemic infection, which would most likely be necessary for cancer induction (for review see [88]). On the other hand, after subcutaneous inoculation of SV40-contaminated inactivated vaccines, antibody titers were high and remained high or declined slightly over a 3-year follow-up [88]. After 1961, IPV was replaced in many countries by OPV, although a few continued to use IPV and others subsequently reintroduced it. We identified four countries that used only IPV between 1957 and 1963 and recalculated differences in mean pleural cancer rates taking into account the type of vaccine used in the country. However, whether SV40-contaminated OPV or IPV was used in a specific country had no impact on asbestos-corrected (male) or raw (female) pleural cancer rates. Many countries used IPV to a variable extent before mass vaccinations with OPV. Unfortunately, hardly any information about these vaccination programs remains available. Therefore, the level of exposure to IPV could not be exactly determined for every country.

We used two different approaches to analyze the association between SV40 prevalence and pleural cancer mortality in European countries. First, we used data on historical SV40 exposure. However, the infected cohorts are diluted by individuals not vaccinated during the possible time of infection or vaccinated with SV40-free vaccines. Second, we screened the literature for molecular genetic evidence of SV40 in tumor samples from the different countries. The methods applied would detect only a strong carcinogenic or co-carcinogenic effect of SV40 on the pleura (in males aggregated by country) because of the crude adjustments for asbestos consumption, without distinguishing between chrysotile and amphibole asbestos. However, the results of this study, the negative SV40 DNA reports, and the serologic data argue against a major role of SV40 in mesothelioma carcinogenesis.

## Conclusion

Finally, despite all the shortcomings and problems, our comprehensive data collection provides the first account of the diverse usage of different types of poliomyelitis virus vaccines from all available data from European countries. In conjunction with the records obtained from the molecular genetic screening for SV40 DNA in tumor samples derived from the respective populations, it provides the best achievable distribution map available to date on SV40 prevalence in Europe and the basis for future reassessments of epidemiological SV40 data, whenever new information becomes available. This ecological analysis makes an association of male pleural cancer with SV40 unlikely, but this needs to be confirmed by case control studies and cohort studies.

## Competing interests

The author(s) declare that they have no competing interests.

## Authors' contributions

K.L., A.L., and O.A. designed the study and edited the manuscript, K.L. and P.R. performed the statistical analysis. H.C. has made substantial contributions to acquisition of data. A.W., R.R., P.K., R.W., and H.O. have been involved in drafting the manuscript or revising the manuscript. All authors read and approved the final manuscript.

## References

[B1] Takahashi K, Huuskonen MS, Tossavainen A, Higashi T, Okubo T, Rantanen J (1999). Ecological relationship between mesothelioma incidence/mortality and asbestos consumption in ten western countries and japan. J Occup Health.

[B2] Tossavainen A (2000). International expert meeting on new advances in the radiology and screening of asbestos-related diseases. Scand J Work Environ Health.

[B3] Peto J, Hodgson JT, Matthews FE, Jones JR (1995). Continuing increase in mesothelioma mortality in Britain. Lancet.

[B4] Carbone M, Pass HI, Miele L, Bocchetta M (2003). New developments about the association of SV40 with human mesothelioma. Oncogene.

[B5] Cerrano PG, Jasani B, Filiberti R, Neri M, Merlo F, De Flora S, Mutti L, Puntoni R (2003). Simian virus 40 and malignant mesothelioma (Review). Int J Oncol.

[B6] Tossavainen A (2004). Global use of asbestos and the incidence of mesothelioma. Int J Occup Environ Health.

[B7] Tossavainen A (1997). Asbestos, asbestosis, and cancer: the Helsinki criteria for diagnosis and attribution. Scand J Work Environ Health.

[B8] McDonald JC, McDonald AD (1996). The epidemiology of mesothelioma in historical context. Eur Respir J.

[B9] Baas P, Schouwink H, Zoetmulder FA (1998). Malignant pleural mesothelioma. Ann Oncol.

[B10] Virta RL, Survey USG (2003). Worldwide asbestos supply and consumption trends from 1900-2000.

[B11] Segura O, Burdorf A, Looman C (2003). Update of predictions of mortality from pleural mesothelioma in the Netherlands. Occup Environ Med.

[B12] Carbone M, Rizzo P, Pass HI (1997). Simian virus 40, poliovaccines and human tumors: a review of recent developments. Oncogene.

[B13] Bocchetta M, Di Resta I, Powers A, Fresco R, Tosolini A, Testa JR, Pass HI, Rizzo P, Carbone M (2000). Human mesothelial cells are unusually susceptible to simian virus 40-mediated transformation and asbestos cocarcinogenity. Proc Natl Acad Sci U S A.

[B14] Cristaudo A, Powers A, Vivaldi A, Foddis R, Guglielmi G, Gattini V, Buselli R, Sensales G, Ciancia E, Ottenga F (2000). SV40 can be reproducibly detected in paraffin-embedded mesothelioma samples. Anticancer Res.

[B15] Cutrone R, Lednicky J, Dunn G, Rizzo P, Bocchetta M, Chumakov K, Minor P, Carbone M (2005). Some oral poliovirus vaccines were contaminated with infectious SV40 after 1961. Cancer Res.

[B16] Gazdar AF, Butel JS, Carbone M (2002). Opinion: SV40 and human tumours: myth, association or causality?. Nat Rev Cancer.

[B17] De Luca A, Baldi A, Esposito V, Howard CM, Bagella L, Rizzo P, Caputi M, Pass HI, Giordano GG, Baldi F, Carbone M, Giordano A (1997). The retinoblastoma gene family pRb/p105, p107, pRb2/p130 and simian virus-40 large T-antigen in human mesotheliomas. Nat Med.

[B18] Shivapurkar N, Takahashi T, Reddy J, Zheng Y, Stastny V, Collins R, Toyooka S, Suzuki M, Parikh G, Asplund S, Kroft SH, Timmons C, McKenna RW, Feng Z, Gazdar AF (2004). Presence of simian virus 40 DNA sequences in human lymphoid and hematopoietic malignancies and their relationship to aberrant promoter methylation of multiple genes. Cancer Res.

[B19] Hirvonen A, Mattson K, Karjalainen A, Ollikainen T, Tammilehto L, Hovi T, Vainio H, Pass HI, Di Resta I, Carbone M, Linnainmaa K (1999). Simian virus 40 (SV40)-like DNA sequences not detectable in finnish mesothelioma patients not exposed to SV40-contaminated polio vaccines. Mol Carcinog.

[B20] De Rienzo A, Tor M, Sterman DH, Aksoy F, Albelda SM, Testa JR (2002). Detection of SV40 DNA sequences in malignant mesothelioma specimens from the United States, but not from Turkey. J Cell Biochem.

[B21] Leithner A, Weinhaeusel A, Windhager R, Schlegl R, Waldner P, Lang S, Dominkus M, Zoubek A, Popper HH, Haas OA (2002). Absence of SV40 in Austrian tumors correlates with low incidence of mesotheliomas. Cancer Biol Ther.

[B22] Krainer M, Schenk T, Zielinski CC, Muller C (1995). Failure to confirm presence of SV40 sequences in human tumours. Eur J Cancer.

[B23] Ohgaki H, Huang H, Haltia M, Vainio H, Kleihues P (2000). More about: cell and molecular biology of simian virus 40: implications for human infections and disease [letter; comment]. J Natl Cancer Inst.

[B24] Emri S, Kocagoz T, Olut A, Gungen Y, Mutti L, Baris YI (2000). Simian virus 40 is not a cofactor in the pathogenesis of environmentally induced malignant pleural mesothelioma in Turkey [see comments]. Anticancer Res.

[B25] Carter JJ, Madeleine MM, Wipf GC, Garcea RL, Pipkin PA, Minor PD, Galloway DA (2003). Lack of serologic evidence for prevalent simian virus 40 infection in humans. J Natl Cancer Inst.

[B26] Engels EA, Viscidi RP, Galloway DA, Carter JJ, Cerhan JR, Davis S, Cozen W, Severson RK, De Sanjose S, Colt JS, Hartge P (2004). Case-control study of simian virus 40 and non-Hodgkin lymphoma in the United States. J Natl Cancer Inst.

[B27] Viscidi RP, Rollison DE, Viscidi E, Clayman B, Rubalcaba E, Daniel R, Major EO, Shah KV (2003). Serological cross-reactivities between antibodies to simian virus 40, BK virus, and JC virus assessed by virus-like-particle-based enzyme immunoassays. Clin Diagn Lab Immunol.

[B28] Knowles WA, Pipkin P, Andrews N, Vyse A, Minor P, Brown DW, Miller E (2003). Population-based study of antibody to the human polyomaviruses BKV and JCV and the simian polyomavirus SV40. J Med Virol.

[B29] Brandner G, Burger A, Neumann-Haefelin D, Reinke C, Helwig H (1977). Isolation of simian virus 40 from a newborn child. J Clin Microbiol.

[B30] Gellrich S, Schewe C, Sterry W, Lukowsky A (2005). Absence of SV40 and other polyomavirus (JCV, BKV) DNA in primary cutaneous B cell lymphomas. J Invest Dermatol.

[B31] Bofill-Mas S, Pina S, Girones R (2000). Documenting the epidemiologic patterns of polyomaviruses in human populations by studying their presence in urban sewage. Appl Environ Microbiol.

[B32] Melnick JL, Stinebaugh S (1962). Excretion of vacuolating SV-40 virus (papova virus group) after ingestion as a contaminant of oral poliovaccine. Proc Soc Exp Biol Med.

[B33] Levi F, Lucchini F, Negri E, Boyle P, La Vecchia C (2004). Cancer mortality in Europe, 1995-1999, and an overview of trends since 1960. Int J Cancer.

[B34] Neuberger M, Vutuc C (2003). Three decades of pleural cncer and mesothelioma registry in Austria where asbestos cement was invented. Int Arch Occup Environ Health.

[B35] La Vecchia C, Lucchini F, Negri E, Boyle P, Maisonneuve P, Levi F (1992). Trends of cancer mortality in Europe, 1955-1989: II, Respiratory tract, bone, connective and soft tissue sarcomas, and skin. Eur J Cancer.

[B36] Brochez L, E. V, Bleyen L, De Backer G, De Bacquer D, Haelterman M, Naeyaert JM (2000). Cancer registration in Belgium: experience from a melanoma registration programme in the province of East-Flanders.. Arch Public Health.

[B37] Metintas S, Metintas M, Ucgun I, Oner U (2002). Malignant mesothelioma due to environmental exposure to asbestos: follow-up of a Turkish cohort living in a rural area. Chest.

[B38] Dhaene K, Verhulst A, Van Marck E (1999). SV40 large T-antigen and human pleural mesothelioma. Screening by polymerase chain reaction and tyramine-amplified immunohistochemistry. Virchows Arch.

[B39] Ramael M, Nagels J, Heylen H, De Schepper S, Paulussen J, De Maeyer M, Van Haesendonck C (1999). Detection of SV40 like viral DNA and viral antigens in malignant pleural mesothelioma. Eur Respir J.

[B40] Hubner R, Van Marck E (2002). Reappraisal of the strong association between simian virus 40 and human malignant mesothelioma of the pleura (Belgium). Cancer Causes Control.

[B41] Brouchet L, Valmary S, Dahan M, Didier A, Galateau-Salle F, Brousset P, Degano B (2005). Detection of oncogenic virus genomes and gene products in lung carcinoma. Br J Cancer.

[B42] Galateau-Salle F, Bidet P, Iwatsubo Y, Gennetay E, Renier A, Letourneux M, Pairon JC, Moritz S, Brochard P, Jaurand MC, Freymuth F (1998). SV40-like DNA sequences in pleural mesothelioma, bronchopulmonary carcinoma, and non-malignant pulmonary diseases. J Pathol.

[B43] Chauvin C, Suh M, Remy C, Benabid AL (1990). Failure to detect viral genomic sequences of three viruses (herpes simplex, simian virus 40 and adenovirus) in human and rat brain tumors. Ital J Neurol Sci.

[B44] Reuther FJ, Löhler J, Herms J, Hugo HH, Schindler C, Leithäuser F, Melzner I, Möller P, Scheil S (2001). Low incidence of SV40-like sequences in ependymal tumours. J Pathol.

[B45] Weggen S, Bayer TA, von Deimling A, Reifenberger G, von Schweinitz D, Wiestler OD, Pietsch T (2000). Low frequency of SV40, JC and BK polyomavirus sequences in human medulloblastomas, meningiomas and ependymomas. Brain Pathol.

[B46] Heinsohn S, Scholz RB, Weber B, Wittenstein B, Werner M, Delling G, Kempf-Bielack B, Setlak P, Bielack S, Kabisch H (2000). SV40 sequences in human osteosarcoma of German origin. Anticancer Res.

[B47] Carbone M, Rizzo P, Procopio A, Giuliano M, Pass HI, Gebhardt MC, Mangham C, Hansen M, Malkin DF, Bushart G, Pompetti F, Picci P, Levine AS, Bergsagel JD, Garcea RL (1996). SV40-like sequences in human bone tumors. Oncogene.

[B48] Shivapurkar N, Wiethege T, Wistuba II, Milchgrub S, Muller KM, Gazdar AF (2000). Presence of simian virus 40 sequences in malignant pleural, peritoneal and noninvasive mesotheliomas. Int J Cancer.

[B49] Krieg P, Amtmann E, Jonas D, Fischer H, Zang K, Sauer G (1981). Episomal simian virus 40 genomes in human brain tumors. Proc Natl Acad Sci U S A.

[B50] Krieg P, Scherer G (1984). Cloning of SV40 genomes from human brain tumors. Virology.

[B51] Heinsohn S, Golta S, Kabisch H, zur Stadt U (2005). Standardized detection of Simian virus 40 by real-time quantitative polymerase chain reaction in pediatric malignancies. Haematologica.

[B52] Völter C, zur Hausen H, Alber D, de Villiers EM (1997). Screening human tumor samples with a broad-spectrum polymerase chain reaction method for the detection of polyomaviruses. Virology.

[B53] Dörries K, Loeber G, Meixensberger J (1987). Association of polyomaviruses JC, SV40, and BK with human brain tumors. Virology.

[B54] Montesinos-Rongen M, Besleaga R, Heinsohn S, Siebert R, Kabisch H, Wiestler OD, Deckert M (2004). Absence of simian virus 40 DNA sequences in primary central nervous system lymphoma in HIV-negative patients. Virchows Arch.

[B55] Geissler E (1983). SV40 and SV40-like viruses as possible risk factors. Arch Geschwulstforsch.

[B56] Martini F, Lazzarin L, Iaccheri L, Corallini A, Gerosa M, Trabanelli C, Calza N, Barbanti-Brodano G, Tognon M (1998). Simian virus 40 footprints in normal human tissues, brain and bone tumours of different histotypes. Dev Biol Stand.

[B57] Gamberi G, Benassi MS, Pompetti F, Ferrari C, Ragazzini P, Sollazzo MR, Molendini L, Merli M, Magagnoli G, Chiesa F, Gobbi AG, Powers A, Picci P (2000). Presence and expression of the simian virus-40 genome in human giant cell tumors of bone. Genes Chromosomes Cancer.

[B58] Strizzi L, Vianale G, Giuliano M, Sacco R, Tassi F, Chiodera P, Casalini P, Procopio A (2000). SV40, JC and BK expression in tissue, urine and blood samples from patients with malignant and nonmalignant pleural disease. Anticancer Res.

[B59] Procopio A, Strizzi L, Vianale G, Betta P, Puntoni R, Fontana V, Tassi G, Gareri F, Mutti L (2000). Simian virus-40 sequences are a negative prognostic cofactor in patients with malignant pleural mesothelioma. Genes Chromosomes Cancer.

[B60] Martinelli M, Martini F, Rinaldi E, Caramanico L, Magri E, Grandi E, Carinci F, Pastore A, Tognon M (2002). Simian virus 40 sequences and expression of the viral large T antigen oncoprotein in human pleomorphic adenomas of parotid glands. Am J Pathol.

[B61] Martini F, Iaccheri L, Lazzarin L, Carinci P, Corallini A, Gerosa M, Iuzzolino P, Barbanti BG, Tognon M (1996). SV40 early region and large T antigen in human brain tumors, peripheral blood cells, and sperm fluids from healthy individuals. Cancer Res.

[B62] Cristaudo A, Vivaldi A, Sensales G, Guglielmi G, Ciancia E, Elisei R, Ottenga F (1995). Molecular biology studies on mesothelioma tumor samples: preliminary data on H-ras, p21, and SV40. J Environ Pathol Toxicol Oncol.

[B63] Martini F, De Mattei M, Iaccheri L, Lazzarin L, Barbanti-Brodano G, Tognon M, Gerosa M (1995). Human brain tumors and simian virus 40. J Natl Cancer Inst.

[B64] Martini F, Lazzarin L, Iaccheri L, Vignocchi B, Finocchiaro G, Magnani I, Serra M, Scotlandi K, Barbanti-Brodano G, Tognon M (2002). Different simian virus 40 genomic regions and sequences homologous with SV40 large T antigen in DNA of human brain and bone tumors and of leukocytes from blood donors. Cancer.

[B65] Pacini F, Vivaldi A, Santoro M, Fedele M, Fusco A, Romei C, Basolo F, Pinchera A (1998). Simian virus 40-like DNA sequences in human papillary thyroid carcinomas. Oncogene.

[B66] Vivaldi A, Pacini F, Martini F, Iaccheri L, Pezzetti F, Elisei R, Pinchera A, Faviana P, Basolo F, Tognon M (2003). Simian virus 40-like sequences from early and late regions in human thyroid tumors of different histotypes. J Clin Endocrinol Metab.

[B67] Tognon M, Martini F, Iaccheri L, Cultrera R, Contini C (2001). Investigation of the simian polyomavirus SV40 as a potential causative agent of human neurological disorders in AIDS patients. J Med Microbiol.

[B68] Martini F, Dolcetti R, Gloghini A, Iaccheri L, Carbone A, Boiocchi M, Tognon M (1998). Simian-virus-40 footprints in human lymphoproliferative disorders of HIV- and HIV+ patients. Int J Cancer.

[B69] Cristaudo A, Foddis R, Vivaldi A, Buselli R, Gattini V, Guglielmi G, Cosentino F, Ottenga F, Ciancia E, Libener R, Filiberti R, Neri M, Betta P, Tognon M, Mutti L, Puntoni R (2005). SV40 Enhances the Risk of Malignant Mesothelioma among People Exposed to Asbestos: A Molecular Epidemiologic Case-Control Study. Cancer Res.

[B70] Paracchini V, Garte S, Pedotti P, Poli F, Frison S, Taioli E (2005). Molecular Identification of Simian Virus 40 Infection in Healthy Italian Subjects by Birth Cohort. Mol Med.

[B71] Capello D, Rossi D, Gaudino G, Carbone A, Gaidano G (2003). Simian virus 40 infection in lymphoproliferative disorders. Lancet.

[B72] De Mattei M, Martini F, Corallini A, Gerosa M, Scotlandi K, Carinci P, Barbanti-Brodano G, Tognon M (1995). High incidence of BK virus large-T-antigen-coding sequences in normal human tissues and tumors of different histotypes. Int J Cancer.

[B73] De Mattei M, Martini F, Tognon M, Serra M, Baldini N, Barbanti-Brodano G (1994). Polyomavirus latency and human tumors. J Infect Dis.

[B74] Hernandez-Losa J, Fedele CG, Pozo F, Tenorio A, Fernandez V, Castellvi J, Parada C, Ramon y Cajal S (2005). Lack of association of polyomavirus and herpesvirus types 6 and 7 in human lymphomas. Cancer.

[B75] Martorell MA, Julian JM, Calabuig C, Garcia-Garcia JA, Perez-Valles A (2002). Lymphoepithelioma-like carcinoma of the uterine cervix. Arch Pathol Lab Med.

[B76] Priftakis P, Bogdanovic G, Hjerpe A, Dalianis T (2002). Presence of simian virus 40 (SV40) is not frequent in Swedish malignant mesotheliomas. Anticancer Res.

[B77] Huang H, Reis R, Yonekawa Y, Lopes JM, Kleihues P, Ohgaki H (1999). Identification in human brain tumors of DNA sequences specific for SV40 large T antigen. Brain Pathol.

[B78] Pepper C, Jasani B, Navabi H, Wynford-Thomas D, Gibbs AR (1996). Simian virus 40 large T antigen (SV40LTAg) primer specific DNA amplification in human pleural mesothelioma tissue. Thorax.

[B79] Griffiths DJ, Nicholson AG, Weiss RA (1998). Detection of SV40 sequences in human mesothelioma. Dev Biol Stand.

[B80] Ibelgaufts H, Jones KW (1982). Papovavirus-related RNA sequences in human neurogenic tumours. Acta Neuropathol (Berl).

[B81] Wong NA, Rae F, Herriot MM, Mayer NJ, Brewster DH, Harrison DJ (2003). SV40 Tag DNA sequences, present in a small proportion of human hepatocellular carcinomas, are associated with reduced survival. J Clin Pathol.

[B82] Mulatero C, Surentheran T, Breuer J, Rudd RM (1999). Simian virus 40 and human pleural mesothelioma. Thorax.

[B83] MacKenzie J, Wilson KS, Perry J, Gallagher A, Jarrett RF (2003). Association between simian virus 40 DNA and lymphoma in the United Kingdom. J Natl Cancer Inst.

[B84] Mayall F, Barratt K, Shanks J (2003). The detection of Simian virus 40 in mesotheliomas from New Zealand and England using real time FRET probe PCR protocols. J Clin Pathol.

[B85] Perrons CJ, Fox JD, Lucas SB, Brink NS, Tedder RS, Miller RF (1996). Detection of polyomaviral DNA in clinical samples from immunocompromised patients: correlation with clinical disease. J Infect.

[B86] Chumakov MP, Voroshilova MK, Drozdov SG, Dzagurov SG, Lashkevich VA, Mironova LL, Ralph NM, Gagarina AV, Dobrova IN, Ashmarina EE, Shirman GA, Fleer GP, Tolskaya EA, Sokolova IS, Elbert LB, Sinyak KM, Weissfeiler J (1961). Some results of the work on mass immunization of the population in the soviet union with live poliovirus vaccine from Albert S. Sabin's strains.. The control of poliomyelitis by live poliovirus vaccine.

[B87] Chumakov MP, Dzagurov SG, Lashkevich VA, Grachev VP, Mironova LL, Ralf NM, Elbert LB, Chumakov MP (1963). Methods and results of preparing live poliovirus vaccine without SV40 virus. Poliomyelitis and other enterovirus infections.

[B88] Shah K, Nathanson N (1976). Human exposure to SV40: review and comment. Am J Epidemiol.

[B89] Geissler E (1990). SV40 and human brain tumors. Prog Med Virol.

[B90] Levine A, Butel J, Dorries K, Goedert J, Frisque R, Garcea R, Morris A, O'Neill F, Shah K (1998). SV40 as a putative human commensal. Dev Biol Stand.

[B91] Kundratitz K (1962). Akutelle Impfprobleme. Mitt d österr Sanitätsverw.

[B92] Friza F (1962). Organisation und Durchführung der ersten Schutzimpfung gegen Kinderlähmung mit Lebendvakzine nach Sabin in Österreich im Winter 1961-1962. Mitt d österr Sanitätsverw.

[B93] Skovránek V, Weissfeiler J (1961). The organization and results of mass vaccination against poliomyelitis in CSSR. The control of poliomyelitis by live poliovirus vaccine.

[B94] (1960). Live poliovirus vaccination in the USSR, Poland and Czechsolovakia. Chron Wld Hlth Org,.

[B95] Engels EA, Katki HA, Nielsen NM, Winther JF, Hjalgrim H, Gjerris F, Rosenberg PS, Frisch M (2003). Cancer incidence in Denmark following exposure to poliovirus vaccine contaminated with simian virus 40. J Natl Cancer Inst.

[B96] Murdin AD, Barreto L, Plotkin S (1996). Inactivated poliovirus vaccine: past and present experience. Vaccine.

[B97] Belian W, Rademacher I, Weissfeiler J (1961). Vaccination with live poliovirus vaccine in the German Democratic Republic. The control of poliomyelitis by live poliovirus vaccine.

[B98] Thu GO, Hem LY, Hansen S, Moller B, Norstein J, Nokleby H, Grotmol T (2006). Is there an association between SV40 contaminated polio vaccine and lymphoproliferative disorders? An age-period-cohort analysis on Norwegian data from 1953 to 1997. Int J Cancer.

[B99] Minor P, Pipkin P, Jarzebek Z, Knowles W (2003). Studies of neutralising antibodies to SV40 in human sera. J Med Virol.

[B100] (1960). Live poliovirus vaccine. Chron Wld Hlth Org.

[B101] Perez Gallardo F, Valenciano Clavel L, Gabriel Y Galan J (1965). Resultados de la Campana nacional de vacunación antipoliomielitica por via oral en Espana. Rev Sanid Hi Publica.

[B102] Sangar D, Pipkin PA, Wood DJ, Minor PD (1999). Examination of poliovirus vaccine preparations for SV40 sequences. Biologicals.

[B103] De Sanjose S, Shah KV, Domingo-Domenech E, Engels EA, Fernandez DS, Alvaro T, Garcia-Villanueva M, Romagosa V, Gonzalez-Barca E, Viscidi RP (2003). Lack of serological evidence for an association between simian virus 40 and lymphoma. Int J Cancer.

[B104] (1958). Four years of poliomyelitis research. Chron Wld Hlth Org.

[B105] (1960). Poliomyelitis prevention. Chron Wld Hlth Org.

[B106] Barbanti-Brodano G, Martini F, De Mattei M, Lazzarin L, Corallini A, Tognon M (1998). BK and JC human polyomaviruses and simian virus 40: natural history of infection in humans, experimental oncogenicity, and association with human tumors. Adv Virus Res.

[B107] Strickler HD (2001). A multicenter evaluation of assays for detection of SV40 DNA and results in masked mesothelioma specimens. Cancer Epidemiol Biomarkers Prev.

[B108] Testa JR, Carbone M, Hirvonen A, Khalili K, Krynska B, Linnainmaa K, Pooley FD, Rizzo P, Rusch V, Xiao GH (1998). A multi-institutional study confirms the presence and expression of simian virus 40 in human malignant mesotheliomas. Cancer Res.

[B109] Knowles WA, Pipkin P, Andrews N, Vyse A, Minor P, Brown DW, Miller E (2003). Population-based study of antibody to the human polyomaviruses BKV and JCV and the simian polyomavirus SV40. J Med Virol.

[B110] Mann RS, Carroll RB (1984). Cross-reaction of BK virus large T antigen with monoclonal antibodies directed against SV40 large T antigen. Virology.

[B111] Beth E, Cikes M, Schloen L, di Mayorca G, Giraldo G (1977). Interspecies-, species- and type-specific T antigenic determinants of human papovaviruses (JC and BK) and of Simian virus 40. Int J Cancer.

[B112] Garcea RL, Imperiale MJ (2003). Simian virus 40 infection of humans. J Virol.

[B113] Barbanti-Brodano G, Sabbioni S, Martini F, Negrini M, Corallini A, Tognon M (2004). Simian virus 40 infection in humans and association with human diseases: results and hypotheses. Virology.

[B114] Senyigit A, Babayigit C, Gokirmak M, Topcu F, Asan E, Coskunsel M, Isik R, Ertem M (2000). Incidence of malignant pleural mesothelioma due to environmental asbestos fiber exposure in the southeast of Turkey. Respiration.

[B115] Pan XL, Day HW, Wang W, Beckett LA, Schenker MB (2005). Residential proximity to naturally occurring asbestos and mesothelioma risk in california. Am J Respir Crit Care Med.

[B116] (2002). Cancer incidence in five continents. Volume VIII. IARC Sci Publ.

[B117] Rizzo P, Di Resta I, Powers A, Matker CM, Zhang A, Mutti L, Kast WM, Pass H, Carbone M (1998). The detection of simian virus 40 in human tumors by polymerase chain reaction. Monaldi Arch Chest Dis.

[B118] Lopez-Rios F, Illei PB, Rusch V, Ladanyi M (2004). Evidence against a role for SV40 infection in human mesotheliomas and high risk of false-positive PCR results owing to presence of SV40 sequences in common laboratory plasmids. Lancet.

[B119] Carbone M, Rdzanek MA, Rudzinski JJ, De Marco MA, Bocchetta M, Ramos NM, Mossman B, Pass HI (2005). SV40 detection in human tumor specimens. Cancer Res.

[B120] Roushdy-Hammady I, Siegel J, Emri S, Testa JR, Carbone M (2001). Genetic-susceptibility factor and malignant mesothelioma in the Cappadocian region of Turkey. Lancet.

[B121] Jasani B, Cristaudo A, Emri SA, Gazdar AF, Gibbs A, Krynska B, Miller C, Mutti L, Radu C, Tognon M, Procopio A (2001). Association of SV40 with human tumours. Semin Cancer Biol.

[B122] Morelli C, Barbisan F, Iaccheri L, Tognon M (2004). SV40-immortalized human fibroblasts as a source of SV40 infectious virions. Mol Med.

